# Does Robotic Roux-en-Y Gastric Bypass Provide Outcome Advantages over Standard Laparoscopic Approaches?

**DOI:** 10.1007/s11695-018-3228-6

**Published:** 2018-04-10

**Authors:** Tomasz Rogula, Marijan Koprivanac, Michał Robert Janik, Jacob A. Petrosky, Amy S. Nowacki, Agnieszka Dombrowska, Matthew Kroh, Stacy Brethauer, Ali Aminian, Philip Schauer

**Affiliations:** 1 University Hospital Cleveland Medical Center/Case Western Reserve University School of Medicine, Cleveland, OH USA; 2 Faculty of Medicine and Health Sciences, Jan Kochanowski University, Kielce, Poland; 3 Bariatric and Metabolic Institute, Cleveland Clinic, Cleveland, OH USA; 4 Department of General, Oncologic, Metabolic and Thoracic Surgery, Military Institute of Medicine, Szaserów 128, 04-141 Warszawa, Poland; 5 Department of Quantitative Health Sciences in the Lerner Research Institute, Cleveland Clinic, Cleveland, OH USA

**Keywords:** Morbid obesity, Bariatric surgery, Robotic, Roux-en-Y gastric bypass, Robot assisted, Gastric bypass, Surgical outcomes, Complications, Anastomotic leak

## Abstract

**Objective:**

The aim was to compare clinical outcomes of patients treated with totally robotic Roux-en-Y gastric bypass (TRRYGB) with those treated with the different laparoscopic Roux-en-Y gastric bypass (LRYGB) techniques.

**Summary Background Data:**

The clinical benefit of the robotic approach to bariatric surgery compared to the standard laparoscopic approach is unclear. There are no studies directly comparing outcomes of TRRYGB with different LRYGB techniques.

**Methods:**

Outcomes of 578 obese patients who underwent RYGB between 2011 and 2014 at an academic center were assessed. Multivariable analysis and propensity matching were used for comparing TRRYGB to different LRYGB techniques, including 21-mm EEA circular-stapled gastrojejunal anastomosis (GJA, LRYGB-21CS), linear-stapled GJA (LRYGB-LS), and hand-sewn GJA (LRYGB-HS).

**Results:**

The TRRYGB technique required a longer mean operative time compared to the other groups, respectively 204 ± 46 vs. 139 ± 30 min (LRYGB-21CS), 206 ± 37 vs. 158 ± 30 min (LRYGB-LS), and 210 ± 36 vs. 167 ± 30 min (LRYGB-HS). TRRYGB experienced a lower stricture rate (2 vs. 17%, *P* = 0.003), shorter hospital stay (2.6 ± 1.2 vs. 4.3 ± 5.5 days, *P* = 0.008), and lower readmission rate (12 vs. 28%, *P* = 0.009). No significant differences in outcomes were observed when comparing RRYGB to LRYGB-LS or LRYGB-HS.

**Conclusions:**

TRRYGB increases operative time compared to all LRYGB techniques. TRRYGB was superior to LRYGB-21CS in terms of significantly shorter hospital stay, lower readmission rate, and less frequent GJA stricture formation. TRRYGB provides no clinical advantages over the LRYGB-LS and LRYGB-HS techniques.

**Electronic supplementary material:**

The online version of this article (10.1007/s11695-018-3228-6) contains supplementary material, which is available to authorized users.

## Introduction

Laparoscopic Roux-en-Y gastric bypass (LRYGB) is one of the most popular bariatric procedures [1]. It has been proven to be effective in achieving and maintaining weight loss [2]. Multiple surgical techniques for LRYGB are performed globally. The main difference is the method for creation of gastrojejunal anastomosis (GJA). Currently, there are three generally accepted laparoscopic GJA techniques, including hand-sewn (HS), linear-stapled (LS), and circular-stapled assisted (CS) technique [3, 4]. Direct comparisons of outcomes of these approaches are limited.

Robotic bariatric surgery has been introduced with the intent to overcome some limitations of laparoscopic methods and specifically improve upon outcomes related to the GJA in RYGB [5]. A robotic GJA (R-GJA) technique involving robotic suturing is commonly utilized. The operation can be performed with total implementation of the robot for the whole procedure (totally robotic RYGB, TRRYGB) or for the GJA creation part (robotic-assisted RYGB, RA-RYGB) [6]. Recently, more surgeons at academic and community practices have incorporated robotic approaches in RYGB and published their outcomes [7–10].

The aim of this study was to compare clinical outcomes of patients treated with totally robotic Roux-en-Y gastric bypass (TRRYGB) with those treated with the various LRYGB techniques.

## Methods

### Selection of Patients

**Fig. 1:**
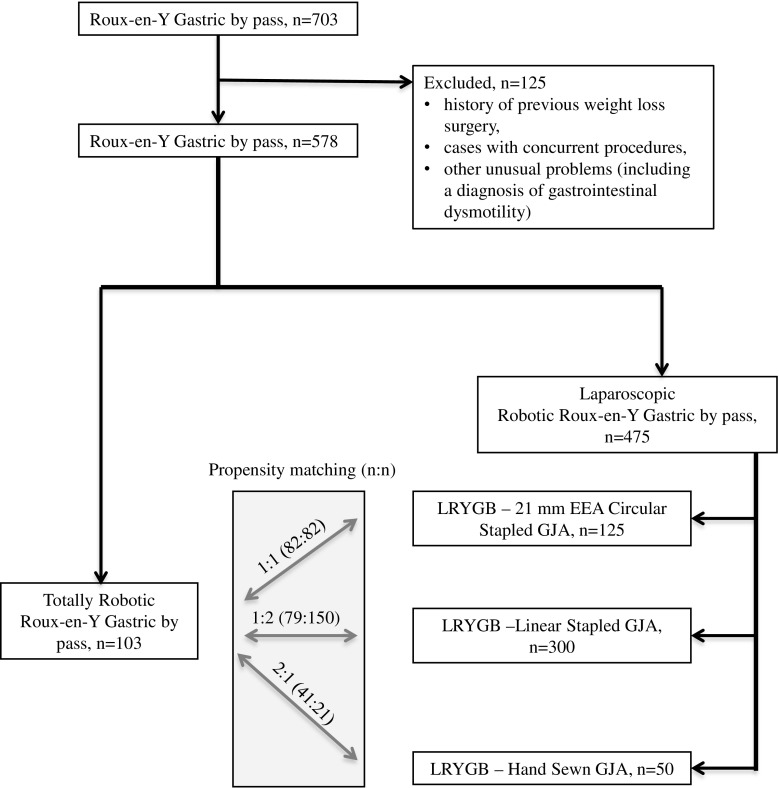
Study flow diagram

This retrospective study was approved by the Institutional Review Board (IRB) of the Cleveland Clinic. Between January 2008 and December 2015, 703 obese patients underwent RYGB in Cleveland Clinic’s Bariatric and Metabolic Institute. All procedures were performed by high-volume (500+ cases/surgeon) bariatric surgeons (TR, MK SB, PS). Study data were reviewed and recorded using an (REDCap) electronic data capture tool hosted by Cleveland Clinic [11]. The exclusion criteria were as follows: history of previous weight loss surgery, any cases with concurrent procedures, or other unusual problems including a diagnosis of gastrointestinal dysmotility (gastroparesis, global bowel dysmotility, colonic inertia). One hundred twenty-five patients were excluded from the study. Five hundred seventy-eight patients were divided into four groups according to technique used for GJA creation: robotic (TRRYGB), 21-mm EEA circular-stapled (LRYGB-21CS), linear-stapled (LRYGB-LS), hand-sewn (LRYGB-HS) (Fig. 1). The patient variables collected included: age, gender, race, initial BMI, and comorbidities (chronic obstructive pulmonary disease, asthma, obstructive sleep apnea, pulmonary hypertension, pulmonary embolism, dyslipidemia, hypertension, diabetes mellitus, coronary artery disease, myocardial infarction, valvular heart disease, cardiomyopathy, cardiac arrhythmia, congestive heart disease, arthritis, metabolic syndrome, tobacco abuse, chronic kidney disease, dialysis, gastroesophageal reflux). In addition, we analyzed preoperative laboratory parameters: creatinine, blood urea nitrogen, glomerular filtration rate, hemoglobin, arterial blood pressure, bilirubin, alanine aminotransferase, and aspartate aminotransferase.

### Surgical Technique

Regardless of surgical approach, construction of jejunojejunal anastomosis was similar in all groups. A 60-mm linear stapler was used for division of the jejunum 50 cm distal to ligament of Treitz (LoT), and was used for stapled anastomosis of a 50-cm bilipancreatic limb (BPL) and a 150-cm Roux limb. Closure for the common opening of the jejunojejunal anastomosis (JJ) was completed with the same stapling device for all laparoscopic approaches. With the robotic approach, the enterotomy was hand-sewn closed. Closure of the mesenteric defect was implemented to prevent internal herniation in the TRRYGB, LRYGB-LS, and LRYGB-HS groups. The greater omentum was divided vertically for the placement of an antecolic antegastric Roux limb; a 15–30 ml pouch was formed with a 60-mm or 3.5-mm height linear stapler. Method of the GJ formation is described below. The diameter of gastrojejunostomy was approximately 15–20 mm regardless of surgical technique used. All cases underwent intraoperatively endoscopy to rule out leaks and to verify the anastomosis diameter.

To make the GJA by linear stapler, the proximal Roux limb was affixed to the posterior wall of the gastric pouch by a running 2-0 absorbable stitch. After making a small gastrotomy and an enterotomy, a blue load Endo GIA linear stapler (Ethicon, Cincinnati, Ohio, USA) was inserted to create the posterior wall of anastomosis. The common opening was closed in a double-layer hand-sewn fashion. Hand-sewn GJA was created by running fashion with 2-0 sutures using Endo-Stitch device. To make a GJA by a circular stapler, we utilized the C21 (Medtronic, Minneapolis, Minnesota, USA) for creation GJA at the corner of the two first staple lines used to create the gastric pouch.

Robotic surgeries were performed with a da Vinci Si robotic platform by a single surgeon (TR). Trocar placement and initial survey of operating field was completed laparoscopically. The robot was then docked. The primary surgeon operated the robot from a single console. An assistant remained at the bedside to aid with retraction and firing of the laparoscopic staplers. The common enterotomy in JJ anastomosis was closed by two layer running sutures. GJA in this group was robot-sewn with a 2-0 absorbable stitch.

### Outcomes

Medical records were evaluated for operative time, length of hospital stay, 1-year readmission, and the following adverse events: any intraoperative complications, including intraabdominal bleeding, return to the operating room within 1 year after surgery, and early and intermediate complications including GJA leaks, GJA strictures, marginal ulcers, small bowel obstruction, wound infection, ventral/internal hernia, deep venous thrombosis, pulmonary embolism, urinary tract infection, urinary retention, acute kidney injury, pneumonia, vomiting, dehydration, and abdominal pain. Weight loss was expressed as a percentage total weight loss ((%TWL = 100*[(BMI_current_ – BMI_baseline_)/BMI_baseline_])) and a change in BMI (ΔBMI).

### Statistical Analysis

The robotic group was matched, on the basis of propensity scores, with the circular stapler group in a 1:1 ratio, with the linear stapler group in a 1:2 ratio and with the hand-sewn group in a 2:1 ratio [12]. These ratios were selected to maximize the matched sample sizes. Propensity scores (i.e., the estimated probability of undergoing robotic surgery on the basis of the preoperative and baseline laboratory values listed above) were estimated for each patient by use of logistic regression. All the pre-specified potential confounding variables were considered; however, variables were excluded from each propensity score model if either the amount of missing data was unacceptable (race and glomerular filtration rate) or comorbidities present in less than five patients per surgery group. After randomly ordering the observations in the dataset, we used a sequential distance matching algorithm to match, each patient in the robotic group to patient(s) undergoing the alternative surgery types, restricting successful matches to those for whom the propensity scores did not differ by more than 0.05 units. The subsets of matched patients were used for all subsequent analyses. Balance on potential confounding variables between the matched robotic group and the EEA stapler, linear stapler or hand-sewn group were evaluated with standard univariable summary statistics and absolute standardized difference scores (absolute value of difference in means or proportions, divided by a combined estimate of standard deviation among the groups being compared). Variables with an absolute standardized difference score of less than 0.20 were considered adequately balanced [13]. Linear mixed models were developed for estimation of the relationships between type of surgery and total operative time, length of hospital stay, and change in percent total weight loss and change in BMI. Generalized estimating equation models were developed for estimation of the relationships between type of surgery and occurrence of intraoperative complication and postoperative complications within 6 months of surgery. All models accounted for the clustering of the propensity score matched patients and assumed an exchangeable correlation structure. A significance level of 0.05 was employed. SAS statistical software version 9.3 (SAS Institute, Cary, NC, USA) for 64-bit Microsoft Windows machine was used for all statistical analyses.

## Results

Study flowchart is shown in Fig. 1. Five hundred seventy-eight patients were included in this study and underwent TRRYGB (*n* = 103) or LRYGB (*n* = 475). LRYGB included 21-mm EEA circular-stapled (21CS, *n* = 125), linear-stapled (LS, *n* = 300), hand-sewn (HS, *n* = 50) groups (Fig. 1).

### TRRYGB Versus LRYGB-21CS

After propensity score matching, the median follow-up was 193 (57, 362) days in TRRYGB and 368 (174, 719) days in LRYGB-21CS group (*P* < 0.001). The mean age of patients in TRRYGB group (*n* = 82) was 43.0 ± 11.3 years, and the mean age of patients after LRYGB-21CS (*n* = 82) was 42.0 ± 11.8 years. The mean BMI in TRRYGB group was 48.5 ± 7.0, whereas the mean BMI in LRYGB-21CS group was 48.0 ± 7.0. There was no statistical difference between groups regarding comorbidities, laboratory results, and smoking status (Supplemental Table 1).

Total operative time was significantly longer in TRRYGB group (204 ± 36 min. versus 139 ± 30, *P* < 0.001). The rate of intraoperative complications was 0% in TRRYGB and 1% in LRYGB-21CS and included intraabdominal bleeding. The mean length of hospital stay was significantly shorter in TRRYGB group in comparison to LRYGB-21CS group (2.6 ± 1.2 vs. 4.3 ± 5.5 days, *P* = 0.008). The readmission rate was significantly lower in TRRYGB group (12 vs. 28%, *P* = 0.009). TRRYGB patients reported less vomiting episodes then those after LRYGB-21CS (15 vs. 33%, *P* = 0.005). The incidence of anastomosis stricture was significantly lower in TRRYGB patients (2 vs. 17%, *P* = 0.003). The mean %TWL after 1 year was significantly lower in TRRYGB patients (− 20.7 ± 10.6 vs. − 25.1 ± 10.6, *P* = 0.02). The mean BMI change after surgery was significantly lower in TRRYGB patients (− 10.3 ± 5.3 vs. − 12.3 ± 5.2, *P* = 0.01). The incidence of marginal ulcer was not statistically significant (12 vs. 23%, *P* = 0.09). The incidence of marginal ulcer was not statistically significant, but possibly clinically important (12 vs. 23%, *P* = 0.09) (Table 1).

**Table 1:** Outcome comparison among TRRYGB and LRYGB-21CS groups after propensity score matching (1:1)

	TRRYGB	LRYGB-21CS	P values^a^
Number of patients	82	82	
Median follow-up, days (Q1, Q3)	193 (57, 362)	368 (174, 719)	< 0.001
Total operative time, minutes	204 ± 36	139 ± 30	< 0.001
Length of hospital stay, days	2.6 ± 1.2	2.6 ± 1.2	0.008
Intraoperative complications	0 (0%)	1 (1%)	–
1 year ΔBMI, kg/m^2^	− 10.0 ± 5.3	− 12.3 ± 5.2	0.01
1 year % TWL	− 20.7 ± 10.6	− 25.1 ± 10.6	0.02
Readmission	10 (12%)	23 (28%)	0.009
Marginal ulcer	10 (12%)	19 (23%)	0.09
Small bowel obstruction	0 (0%)	5 (6%)	–
Ventral hernia	1 (1%)	0 (0%)	–
Internal hernia	1 (1%)	2 (2%)	0.56
Pulmonary embolism	0 (0%)	1 (1%)	–
Deep vein thrombosis	0 (0%)	0 (0%)	–
Intraabdominal bleeding	1 (1%)	5 (6%)	0.10
GJA leak	0 (0%)	2 (2%)	–
GJA stricture	2 (2%)	14 (17%)	0.003
Reoperation	5 (6%)	5 (6%)	0.99
Acute kidney injury	0 (0%)	0 (0%)	–
Wound infection	2 (2%)	5 (6%)	0.26
Intraabdominal infection	4 (5%)	1 (1%)	0.18
Pneumonia	1 (1%)	2 (2%)	0.56
Urinary tract infection	1 (1%)	3 (4%)	0.32
Urinary retention	0 (0%)	0 (0%)	–
Vomiting	12 (15%)	27 (33%)	0.005
Dehydration	11 (13%)	14 (17%)	0.49

^a^P values result from either general linear mixed model or McNemar’s test

### TRRYGB Versus LRYGB-LS

After propensity score matching, the median follow-up was 205 (57, 365) days in TRRYGB and 299 (135, 589) days in LTYGB-LS group (*P* < 0.001). The mean age of patients in TRRYGB group (*n* = 79) was 43.4 ± 11.9 years, and the mean age those who underwent LRYGB-LS (*n* = 150) was 44.3 ± 12.0 years. The mean BMI of patients in TRRYGB group was 48.7 ± 7.2, whereas the mean BMI in LRYGB-LS group was 48.5 ± 7.0. There was no statistical difference between groups regarding comorbidities, laboratory results, and smoking status (Supplemental Table 2).

Total operative time was significantly longer in TRRYGB group (206 ± 37 min vs. 158 ± 30 min, *P* < 0.001). The rate of intraoperative complications was 0% in TRRYGB and 2% in LRYGB-LS. There was no statistical difference in mean length of hospital stay in TRRYGB (2.5 ± 1.1 vs. 2.8 ± 1.0 days, *P* − 0.06). The readmission rate was comparable between TRRYGB and LRYGB-LS (13 vs. 11%, *P* = 0.64). No statistical difference was observed in the incidence of anastomosis leak, anastomosis stricture, and other complications. There was no significant difference in %TWL and ΔBMI at the 1-year follow-up (Table 2).

**Table 2:** Outcome comparison among TRRYGB and LRYGB-LS groups after propensity score matching (1:2)

	TRRYGB	LRYGB-LS	P values^a^
Number of patients	79	150	
Median follow-up, days (Q1, Q3)	205 (57, 365)	299 (135, 589)	< 0.001
Total operative time, minutes	206 ± 37	158 ± 30	< 0.001
Length of hospital stay, days	2.5 ± 1.1	2.8 ± 1.0	0.06
Intraoperative complications	0 (0%)	3 (2%)	–
1 year ΔBMI, kg/m^2^	− 10.0 ± 5.6	− 10.7 ± 5.6	0.34
1 year % TWL	− 20.5 ± 11.0	− 21.8 ± 10.5	0.37
Readmission	10 (13%)	16 (11%)	0.64
Marginal ulcer	10 (13%)	4 (9%)	0.47
Small bowel obstruction	0 (0%)	0 (0%)	–
Ventral hernia	1 (1%)	0 (0%)	–
Internal hernia	1 (1%)	0 (0%)	–
Pulmonary embolism	0 (0%)	0 (0%)	–
Deep vein thrombosis	0 (0%)	0 (0%)	–
Intraabdominal bleeding	1 (1%)	0 (0%)	–
GJA leak	0 (0%)	0 (0%)	–
GJA stricture	2 (3%)	4 (3%)	0.95
Reoperation	5 (6%)	2 (1%)	0.09
Acute kidney injury	0 (0%)	0 (0%)	–
Wound infection	2 (3%)	2 (1%)	0.56
Intraabdominal infection	4 (5%)	0 (0%)	–
Pneumonia	1 (1%)	0 (0%)	–
Urinary tract infection	1 (1%)	5 (3%)	0.29
Urinary retention	0 (0%)	1 (1%)	–
Vomiting	12 (15%)	27 (18%)	0.60
Dehydration	12 (15%)	12 (8%)	0.13

^a^P values result from either general linear mixed model or McNemar’s test

### TRRYGB Versus LRYGB-HS

After propensity score matching, the median follow-up was 198 (84, 365) days in TRRYGB and 214 (114, 372) days in LRYGB-HS group (*P* = 0.11). The mean age of patients in TRRYGB group (*n* = 41) was 46.0 ± 12.3 years, and the mean age in LRYGB-HS group (*n* = 21) was 46.5 ± 13.6 years (*P* = 0.89). The mean BMI of patients in the TRRYGB group was 46.3 ± 7.4, whereas the mean BMI in LRYGB-HS group was 45.4 ± 5.6 (*P* = 0.66). There were no differences in comorbidities, laboratory results, and smoking status (Supplemental Table 3).

Total operative time was significantly longer in TRRYGB group (210 ± 36 min vs. 167 ± 30 min, *P* < 0.001). The rate of intraoperative complications was 2% in TRRYGB and 10% in LRYGB-LS but based on small sample sizes (*P* = 0.32). There was no difference in mean length of hospital stay in TRRYGB (2.9 ± 1.6 vs. 3.6 ± 2.9 days, *P* = 0.25).The readmission rate was comparable between TRRYGB and LRYGB-HS groups (15 vs. 14%, *P* = 0.97). No statistical differences were observed in the incidence of anastomosis leak, anastomosis stricture, and other complications. There was no significant difference in %TWL and ΔBMI at the 1-year follow-up (Table 3).

**Table 3:** Outcome comparison among TRRYGB and LRYGB-HS groups after propensity score matching (2:1)

	TRRYGB	LRYGB-HS	P values^a^
Number of patients	41	21	
Median follow-up, days (Q1, Q3)	198 (84, 365)	214 (114, 372)	0.11
Total operative time, minutes	210 ± 36	167 ± 30	< 0.001
Length of hospital stay, days	2.9 ± 1.6	3.6 ± 2.9	0.25
Intraoperative complications	1 (2%)	2 (10%)	0.32
1 year ΔBMI, kg/m^2^	− 9.8 ± 5.3	− 10.4 ± 5.2	0.71
1 year % TWL	− 21.2 ± 11.4	− 21.7 ± 10.0	0.87
Readmission	6 (15%)	3 (14%)	0.97
Marginal ulcer	6 (15%)	4 (19%)	0.69
Small bowel obstruction	0 (0%)	0 (0%)	–
Ventral hernia	0 (0%)	0 (0%)	–
Internal hernia	1 (2%)	0 (0%)	–
Pulmonary embolism	0 (0%)	0 (0%)	–
Deep vein thrombosis	0 (0%)	1 (5%)	–
Intraabdominal bleeding	1 (2%)	1 (5%)	0.67
GJA leak	1 (2%)	0 (0%)	–
GJA stricture	0 (0%)	2 (10%)	–
Reoperation	2 (5%)	0 (0%)	–
Acute kidney injury	0 (0%)	1 (5%)	–
Wound infection	0 (0%)	0 (0%)	–
Intraabdominal infection	1 (2%)	1 (5%)	0.67
Pneumonia	1 (2%)	2 (10%)	0.32
Urinary tract infection	0 (0%)	1 (5%)	–
Urinary retention	0 (0%)	0 (0%)	–
Vomiting	6 (15%)	2 (10%)	0.55
Dehydration	8 (20%)	2 (10%)	0.36

^a^P values result from either general linear mixed model or McNemar’s test

## Discussion

This is the first study, utilizing propensity matching to compare the outcomes of totally robotic RYGB to different laparoscopic RYGB techniques with variations on construction of the GJA. Between 31 and 36 variables were used in the propensity matching of cohorts in an attempt to obtain highly comparable groups. The comparison of clinical outcomes was made in an effort to determine advantages and disadvantages of TRRYGB.

This study revealed longer operative time in TRRYGB group in comparison to the all LRYGB techniques. This was the only significant difference in outcomes including postoperative complication rates between TRRYGB and LRYGB-LS, as well as between TRRYGB and LRYGB-HS group. During the study period, we attempted to reduce the operating times with the robot. The attempt included mobilization of the stomach and creation of the gastric pouch by standard laparoscopy and firing of the stapler prior to docking of the robot. Despite any theoretical efficiencies gained by use of the same robotic scrub team, we were unable to reduce operative room times to that comparable with laparoscopic techniques.

An important finding of this study is the higher rate of endoscopically verified anastomosis stricture in LRYGB-21CS group (17%), in comparison to patients after TRRYGB (2%); *P* = 0.003. No difference in stricture rate was seen with the hand-sewn or linear stapler technique suggesting that the narrow 21-mm circular stapler likely accounts for the higher stricture rate.

All strictures were detected clinically and confirmed endoscopically. A standard balloon pneumatic dilatation was used as a treatment. None of the patient in LRYGB-21CS group required anastomotic revision due to stricture. Symptomatic patients with no obvious strictures in endoscopy were treated conservatively with gradually improving symptoms. These patients suffered mainly from anastomotic edema in early postoperative period. Many series have shown that the use of a 21-mm circular stapler is associated with higher rates of stricture. Our 17% stricture rate with the 21-mm circular stapler is consistent with previous reports revealing high stricture rates in the range of 12.5 to 27% [3, 14, 15]. Sima et al. reported a greater incidence of vomiting and need for endoscopic dilation over a 5-year follow-up period in those patients for whom the C21 technique was used for GJA [14]. However, some authors have demonstrated low rates of GJ stricture with the 21-mm staplers (0.8–5.9%) [16, 17]. Factors associated with formation of stricture at the GJA include a 21-mm diameter CS and a 4.5-mm staple height. When using the C21, the rate of stricture formation and hemorrhagic complications was reduced by moving away from 4.5-mm height staples and using 3.5-mm height staples for the GJA [18–20].

In response to our findings, we currently employ LRYGB-LS and LRYGB-HS methods, or the 25-mm diameter CS for creation of the GJA.

Longer hospital stay in LRYGB-21CS group was due to wound problems at the site of insertion of circular stapler (left upper quadrant); some of these patients required extensive pain control and few developed superficial wound infection. What is more, some patients experienced nausea and vomiting, likely due to early postoperative anastomotic edema, which required prolonged intra venous fluid infusion and symptomatic treatment with antimemetics.

Since both robotic and laparoscopic approaches are minimally invasive and equally reduce access incisions compared to open surgery, our findings of similar postoperative complication rates between the two approaches are not surprising. However, proponents of robotic surgery have argued that increased operative precision compared to laparoscopic surgery leads to reduced surgical complications, such as leaks, bleeding, obstructions, venous thromboembolism, and infections. Such an advantage in precision of the robotic approach has not thus far been supported by others. In one systematic review of 27 observational studies (27,997 patients), Li K et al. found no difference in overall complications, major complications, mortality, reoperations, or length of stay between robotic and laparoscopic RYGB, but did find longer operating time and higher cost with the robotic approach [21]. A meta-analysis by Economopoulos et al. (5145 patients) revealed no difference in anastomotic leaks and strictures between robotic and laparoscopic techniques [6]. In yet another review of 27 studies, Bindal et al. found no consistent advantage of the robotic approach over laparoscopic approach for RYGB [22].

The readmission rate was significantly lower in TRRYGB group in comparison to LRYGB-21CS. The difference was not observed in case of LRYGB-LS and LRYGB-HS.

The reasons for readmission were following: in TRRYGB—marginal ulcers (including one perforated), internal hernia, dehydration due to nausea and vomiting; in LRYGB-21CS—nausea and vomiting due to anastomotic strictures and marginal ulcers, small bowel obstruction, internal hernia, wound infections, uncontrolled pain, and pulmonary embolism; in LRYGB-LS—marginal ulcers, small bowel obstruction, pneumonia, dehydration due to nausea and vomiting; in LRYGB-LS—marginal ulcers, dehydration, and pneumonia.

We found a significant difference in %TWL 1 year after surgery between TRRYGB (− 20.7%) and LRYGB-21CS (− 25.1%) *P* = 0.02, favoring LRYGB-21CS. The difference was not present in comparison of TRRYGB and other LRYGB techniques. Possibly, the narrow stoma resulting from the 21-mm stapler accounts for the improved weight loss. However, it should be stressed out that due to the difference in median follow-up time this result is inconclusive. Currently, there were only two studies reporting weight change in comparing the robotic and laparoscopic RYGB technique [23, 24]. None of them reported significant differences.

Limitations of the present study include the fact that it was retrospective, non-randomized, and conducted at a single academic institution. To account for variance in patient characteristics, propensity matching was used but also reduced the number of patients matched in each cohort. These results should not be extrapolated to long-term outcomes; by design, we have looked at early outcomes and early postoperative complications. The small sample size can affect generalizability. Outcomes following use of the C21 cannot be generalized to techniques performed with the C25 method for GJA and will require further study. Lastly, a significant difference in length of follow-up was present when comparing TRRYGB to LRYGB-21CS and to LRYGB-LS. However, the majority of assessed complications, including GJA stricture was typical for the early postoperative period. The 30-day follow-up was completed in all cases.

## Conclusions

The robotic approach to RYGB resulted in no reduction in postoperative complications compared to laparoscopic approaches utilizing hand-sewn or linear stapler for the gastrojejunal anastomosis. The LRYGB with the 21-mm circular stapler had an inordinately high stricture rate of 17% that likely contributed to longer hospital stay and higher readmission rate compared to the robotic approach. The robotic RYGB required significantly longer operative times than each of the alternate laparoscopic techniques. These findings suggest that totally robotic RYGB currently provides no clinical advantages over laparoscopic techniques for RYGB and increases operative time and resources. Randomized controlled trials directly comparing robotic vs. laparoscopic approaches are needed before the robotic approach to RYGB can be considered an alternative to standard laparoscopic approaches.

## Electronic supplementary material

Supplemental Table 1 (DOCX 127 kb)

Supplemental Table 2 (DOCX 121 kb)

Supplemental Table 3 (DOCX 122 kb)
