# Supplementary material for: Does Robotic Roux-en-Y Gastric Bypass Provide Outcome Advantages over Standard Laparoscopic Approaches?
Source: Obes Surg. 2018 Apr 10;28(9):2589–96. doi: 10.1007/s11695-018-3228-6 (PMC6132787; doi:10.1007/s11695-018-3228-6)
Supplement: Supplementary file 1 — (DOCX 127 kb) [file 11695_2018_3228_MOESM1_ESM.docx]

**Supplemental Table 1.** Demographic and clinical characteristic comparison among TRRYGB and LRYGB-21CS groups before and after propensity score matching (1:1).

|  | **Before PS Matching** | | |  | **After PS Matching** | | | |
| --- | --- | --- | --- | --- | --- | --- | --- | --- |
|  | **TRRYGB** | **LRYGB-21CS** | p^b^ |  | **TRRYGB** | **LRYGB-21CS** | p^c^ | |
| Count | 103 | 125 |  |  | 82 | 82 |  | |
| Length of follow-up, days^a^ | 196 (65, 378) | 325 (108, 586) | <0.001 |  | 193 (57, 362) | 368 (174,719) | <0.001 | |
|  |  |  |  |  |  |  |  | |
| **Demographics** |  |  |  |  |  |  |  | |
| Age, years | 44.5 ± 11.5 | 44.5 ± 12.4 | 0.99 |  | 43.0 ± 11.3 | 42.0 ±11.8 | 0.50 | |
| Female | 78 (76%) | 92 (74%) | 0.76 |  | 62 (76%) | 62 (76%) | 0.99 | |
| Caucasian | 54 (75%) | 77 (79%) | 0.59 |  | 45 (79%) | 51 (81%) | 0.99 | |
| Body mass index, kg/m^2^ | 48.3 ± 7.0 | 48.5 ± 8.2 | 0.84 |  | 48.5 ± 7.0 | 48.0 ± 7.1 | 0.59 | |
| [Chronic obstructive pulmonary disease](http://en.wikipedia.org/wiki/COPD) | 6 (6%) | 5 (4%) | 0.76 |  | 3 (4%) | 1 (1%) | 0.32 | |
| Asthma | 19 (19%) | 16 (14%) | 0.36 |  | 15 (18%) | 12 (15%) | 0.51 | |
| Sleep apnea | 66 (67%) | 83 (67%) | 0.99 |  | 56 (68%) | 51 (62%) | 0.38 | |
| Pulmonary hypertension | 1 (1%) | 2 (2%) | 0.99 |  | 1 (1%) | 1 (1%) | 0.99 | |
| Pulmonary emboli | 1 (1%) | 0 (0%) | 0.46 |  | 0 (0%) | 0 (0%) | - | |
| Dyslipidemia | 37 (37%) | 50 (42%) | 0.49 |  | 25 (30%) | 26 (32%) | 0.86 | |
| Hypertension | 51 (50%) | 74 (60%) | 0.18 |  | 37 (45%) | 36 (44%) | 0.87 | |
| Diabetes mellitus I | 1 (1%) | 2 (2%) | 0.99 |  | 0 (0%) | 1 (1%) | - | |
| Diabetes mellitus II | 24 (24%) | 34 (29%) | 0.45 |  | 15 (18%) | 14 (17%) | 0.83 | |
| Diabetes mellitus unspecified | 3 (3%) | 3 (3%) | 0.99 |  | 3 (4%) | 2 (2%) | 0.65 | |
| Coronary artery disease | 10 (10%) | 7 (6%) | 0.32 |  | 7 (8%) | 5 (6%) | 0.48 | |
| Myocardial infarction | 0 (0%) | 0 (0%) | - |  | 0 (0%) | 0 (0%) | - | |
| Valvular heart disease | 2 (2%) | 4 (4%) | 0.69 |  | 2 (2%) | 1 (1%) | 0.56 | |
| Cardiomyopathy | 1 (1%) | 4 (4%) | 0.38 |  | 1 (1%) | 2 (2%) | 0.56 | |
| Cardiac arrhythmia | 8 (8%) | 10 (9%) | 0.99 |  | 6 (7%) | 5 (6%) | 0.76 | |
| Congestive heart failure | 5 (5%) | 6 (5%) | 0.99 |  | 3 (4%) | 5 (6%) | 0.48 | |
| Arthritis | 28 (27%) | 24 (20%) | 0.21 |  | 21 (26%) | 16 (20%) | 0.28 | |
| Metabolic syndrome | 9 (9%) | 7 (6%) | 0.45 |  | 5 (6%) | 6 (7%) | 0.74 | |
| Smoker | 5 (5%) | 18 (15%) | **0.01** |  | 4 (5%) | 2 (2%) | 0.32 | |
| Chronic kidney disease | 3 (3%) | 4 (3%) | 0.99 |  | 2 (2%) | 2 (2%) | 0.99 | |
| Dialysis | 0 (0%) | 1 (1%) | - |  | 0 (0%) | 1 (1%) | - | |
| [Gastroesophageal reflux disease](http://en.wikipedia.org/wiki/Gastroesophageal_reflux_disease) | 36 (36%) | 36 (31%) | 0.47 |  | 26 (32%) | 23 (28%) | 0.59 | |
|  |  |  |  |  |  |  |  | |
| **Labs** |  |  |  |  |  |  |  | |
| Creatinine | 0.86 ± 0.28 | 0.86 ± 0.51 | 0.91 |  | 0.84 ± 0.28 | 0.86 ± 0.59 | 0.79 | |
| [Blood urea nitrogen, mmol/L](http://labtestsonline.org/understanding/analytes/bun/tab/test) | 15.5 ± 6.5 | 15.4 ± 7.2 | 0.90 |  | 15.1 ± 6.3 | 14.8 ± 6.4 | 0.79 | |
| Glomerular filtration rate | 58.1 ± 8.4 | 58.4 ± 8.1 | 0.80 |  | 59.0 ± 4.7 | 59.2 ± 6.1 | 0.82 | |
| Hemoglobin, gm/dL | 13.4 ± 1.4 | 13.6 ± 1.4 | 0.29 |  | 13.4 ±1.4 | 13.5 ± 1.2 | 0.68 | |
| Mean blood pressure, mmHg | 92.0 ± 14.1 | 93.0 ± 10.5 | 0.54 |  | 92.5 ±10.5 | 93.4 ± 11.4 | 0.54 | |
| Bilirubin, mg/dL | 0.45 ± 0.23 | 0.44 ± 0.25 | 0.79 |  | 0.44 ± 0.21 | 0.43 ±0.23 | 0.81 | |
| [Alanine aminotransferase](http://labtestsonline.org/understanding/analytes/alt) | 31.3 ± 34.5 | 30.8 ± 19.1 | 0.88 |  | 33.0 ± 38.0 | 32.3 ±20.7 | 0.89 | |
| Aspartate aminotransferase | 28.8 ± 29.6 | 28.2 ± 16.5 | 0.86 |  | 30.0 ± 32.5 | 29.5 ±18.9 | 0.91 | |
| Descriptive characteristics reported as mean and ± standard deviation or count (%)  ^a^ median (quartile 1 , quartile 3)  ^b^ p-values result from either one-way ANOVA or Fisher's exact test  ^c^ p-values result from linear mixed model or generalized estimating equation | | | | | | | |  |
