# Supplementary material for: Does Robotic Roux-en-Y Gastric Bypass Provide Outcome Advantages over Standard Laparoscopic Approaches?
Source: Obes Surg. 2018 Apr 10;28(9):2589–96. doi: 10.1007/s11695-018-3228-6 (PMC6132787; doi:10.1007/s11695-018-3228-6)
Supplement: Supplementary file 2 — (DOCX 121 kb) [file 11695_2018_3228_MOESM2_ESM.docx]

**Supplemental Table 2. Demographic and clinical characteristic comparison among TRRYGB and LRYGB-LS groups before and after propensity score matching.**

|  | **Before PS Matching** | | |  | **After PS Matching** | | | |
| --- | --- | --- | --- | --- | --- | --- | --- | --- |
|  | **TRRYGB** | **LRYGB-LS** | p^b^ |  | **TRRYGB** | **LRYGB-LS** | p^c^ | |
| Count | 103 | 300 |  |  | 79 | 150 |  | |
| Length of follow-up, days^a^ | 196 (65, 378) | 334 (146, 684) | <0.001 |  | 205 (57,365) | 299 (135,589) | <0.001 | |
|  |  |  |  |  |  |  |  | |
| **Demographics** |  |  |  |  |  |  |  | |
| Age, years | 44.5 ± 11.5 | 44.8 ± 11.5 | 0.83 |  | 43.4 ± 11.9 | 44.3 ± 12.0 | 0.57 | |
| Female | 78 (76%) | 228 (76%) | 0.99 |  | 60 (76%) | 122 (81%) | 0.36 | |
| Caucasian | 54 (75%) | 192 (78%) | 0.53 |  | 44 (80%) | 92 (76%) | 0.57 | |
| Body mass index, kg/m^2^ | 48.3 ± 7.0 | 48.4 ± 8.3 | 0.92 |  | 48.7 ±7.2 | 48.5 ± 7.0 | 0.85 | |
| [Chronic obstructive pulmonary disease](http://en.wikipedia.org/wiki/COPD) | 6 (6%) | 14 (5%) | 0.50 |  | 3 (4%) | 9 (6%) | 0.43 | |
| Asthma | 19 (19%) | 49 (18%) | 0.88 |  | 13 (16%) | 30 (20%) | 0.47 | |
| Sleep apnea | 66 (67%) | 174 (61%) | 0.40 |  | 52 (66%) | 99 (66%) | 0.97 | |
| Pulmonary hypertension | 1 (1%) | 2 (1%) | 0.99 |  | 1 (1%) | 2 (1%) | 0.96 | |
| Pulmonary emboli | 1 (1%) | 4 (2%) | 0.99 |  | 0 (0%) | 1 (1%) | - | |
| Dyslipidemia | 37 (37%) | 133 (47%) | 0.08 |  | 25 (32%) | 53 (35%) | 0.58 | |
| Hypertension | 51 (50%) | 188 (66%) | **0.007** |  | 37 (47%) | 73 (49%) | 0.79 | |
| Diabetes mellitus I | 1 (1%) | 3 (1%) | 0.99 |  | 0 (0%) | 1 (1%) | - | |
| Diabetes mellitus II | 24 (24%) | 109 (39%) | **0.007** |  | 17 (22%) | 42 (28%) | 0.14 | |
| Diabetes mellitus unspecified | 3 (3%) | 7 (3%) | 0.99 |  | 2 (3%) | 4 (3%) | 0.94 | |
| Coronary artery disease | 10 (10%) | 22 (8%) | 0.54 |  | 6 (8%) | 12 (8%) | 0.93 | |
| Myocardial infarction | 0 (0%) | 2 (1%) | 0.99 |  | 0 (0%) | 0 (0%) | - | |
| Valvular heart disease | 2 (2%) | 3 (1%) | 0.62 |  | 1 (1%) | 2 (1%) | 0.96 | |
| Cardiomyopathy | 1 (1%) | 8 (3%) | 0.45 |  | 1 (1%) | 3 (3%) | 0.67 | |
| Cardiac arrhythmia | 8 (8%) | 19 (7%) | 0.66 |  | 4 (5%) | 6 (4%) | 0.71 | |
| Congestive heart failure | 5 (5%) | 9 (3%) | 0.54 |  | 3 (4%) | 4 (3%) | 0.66 | |
| Arthritis | 28 (27%) | 57 (21%) | 0.21 |  | 21 (27%) | 33 (22%) | 0.48 | |
| Metabolic syndrome | 9 (9%) | 48 (18%) | **0.04** |  | 6 (8%) | 18 (12%) | 0.20 | |
| Smoker | 5 (5%) | 45 (17%) | **0.003** |  | 4 (5%) | 10 (7%) | 0.57 | |
| Chronic kidney disease | 3 (3%) | 8 (3%) | 0.99 |  | 2 (3%) | 5 (3%) | 0.72 | |
| Dialysis | 0 (0%) | 3 (1%) | 0.57 |  | 0 (0%) | 1 (1%) | - | |
| [Gastroesophageal reflux disease](http://en.wikipedia.org/wiki/Gastroesophageal_reflux_disease) | 36 (36%) | 74 (27%) | 0.12 |  | 21 (27%) | 43 (29%) | 0.72 | |
|  |  |  |  |  |  |  |  | |
| **Labs** |  |  |  |  |  |  |  | |
| Creatinine | 0.86 ± 0.28 | 0.86 ± 0.37 | 0.89 |  | 0.85 ± 0.29 | 0.84 ± 0.25 | 0.85 | |
| [Blood urea nitrogen, mmol/L](http://labtestsonline.org/understanding/analytes/bun/tab/test) | 15.5 ± 6.5 | 15.6 ± 7.2 | 0.96 |  | 15.3 ± 6.8 | 15.9 ± 8.2 | 0.62 | |
| Glomerular filtration rate | 58.1 ± 8.4 | 58.4 ± 7.0 | 0.79 |  | 0.85 ± 0.29 | 0.84 ± 0.25 | 0.85 | |
| Hemoglobin, gm/dL | 13.4 ± 1.4 | 13.5 ± 1.4 | 0.33 |  | 13.4±1.3 | 13.4 ± 1.4 | 0.92 | |
| Mean blood pressure, mmHg | 92.0 ± 14.1 | 93.4 ± 12.8 | 0.36 |  | 92.6 ± 11.0 | 92.2 ± 12.9 | 0.84 | |
| Bilirubin, mg/dL | 0.45 ± 0.23 | 0.47 ± 0.25 | 0.57 |  | 0.44 ± 0.20 | 0.45 ± 0.23 | 0.59 | |
| [Alanine aminotransferase](http://labtestsonline.org/understanding/analytes/alt) | 31.3 ± 34.5 | 31.7 ± 21.4 | 0.88 |  | 33.0 ± 38.2 | 31.2 ± 20.2 | 0.64 | |
| Aspartate aminotransferase | 28.8 ± 29.6 | 28.2 ± 20.2 | 0.82 |  | 29.7 ± 32.9 | 27.3 ± 13.1 | 0.44 | |
| Descriptive characteristics reported as mean and ± standard deviation or count (%)  ^a^ median (quartile 1 , quartile 3)  ^b^ p-values result from either one-way ANOVA or Fisher's exact test  ^c^ p-values result from linear mixed model or generalized estimating equation | | | | | | | |  |
