# Supplementary material for: Does Robotic Roux-en-Y Gastric Bypass Provide Outcome Advantages over Standard Laparoscopic Approaches?
Source: Obes Surg. 2018 Apr 10;28(9):2589–96. doi: 10.1007/s11695-018-3228-6 (PMC6132787; doi:10.1007/s11695-018-3228-6)
Supplement: Supplementary file 3 — (DOCX 122 kb) [file 11695_2018_3228_MOESM3_ESM.docx]

**Supplemental Table 3. Demographic and clinical characteristic comparison among TRRYGB and LRYGB-HS groups before and after propensity score matching.**

|  | **Before PS Matching** | | |  | **After PS Matching** | | | |
| --- | --- | --- | --- | --- | --- | --- | --- | --- |
|  | **TRRYGB** | **LRYGB-HS** | p^b^ |  | **TRRYGB** | **LRYGB-HS** | p^c^ | |
| Count | 103 | 50 |  |  | 41 | 21 |  | |
| Length of follow-up, days^a^ | 196 (65, 378) | 186 (92, 259) | 0.73 |  | 198 (84, 365) | 214 (111, 372) | 0.11 | |
|  |  |  |  |  |  |  |  | |
| **Demographics** |  |  |  |  |  |  |  | |
| Age, years | 44.5 ± 11.5 | 49.6 ± 11.3 | 0.01 |  | 46.0 ± 12.3 | 46.5 ±13.6 | 0.89 | |
| Female | 78 (76%) | 37 (74%) | 0.84 |  | 32 (78%) | 15 (71%) | 0.52 | |
| Caucasian | 54 (75%) | 27 (77%) | 0.99 |  | 21 (75%) | 11 (69%) | 0.65 | |
| Body mass index, kg/m^2^ | 48.3 ± 7.0 | 44.9 ± 6.0 | **0.003** |  | 46.3 ± 7.4 | 45.4 ± 5.6 | 0.66 | |
| [Chronic obstructive pulmonary disease](http://en.wikipedia.org/wiki/COPD) | 6 (6%) | 1 (2%) | 0.42 |  | 3 (7%) | 1 (1%) | 0.69 | |
| Asthma | 19 (19%) | 12 (24%) | 0.53 |  | 8 (20%) | 4 (19%) | 0.98 | |
| Sleep apnea | 66 (67%) | 34 (68%) | 0.99 |  | 27 (66%) | 14 (67%) | 0.95 | |
| Pulmonary hypertension | 1 (1%) | 1 (2%) | 0.99 |  | 0 (0%) | 0 (0%) | - | |
| Pulmonary emboli | 1 (1%) | 2 (4%) | 0.27 |  | 1 (2%) | 1 (5%) | 0.67 | |
| Dyslipidemia | 37 (37%) | 30 (60%) | **0.009** |  | 17 (41%) | 7 (33%) | 0.46 | |
| Hypertension | 51 (50%) | 31 (62%) | 0.17 |  | 20 (49%) | 11 (52%) | 0.74 | |
| Diabetes mellitus I | 1 (1%) | 0 (0%) | 0.99 |  | 0 (0%) | 0 (0%) | - | |
| Diabetes mellitus II | 24 (24%) | 27 (55%) | **<0.001** |  | 14 (34%) | 7 (33%) | 0.93 | |
| Diabetes mellitus unspecified | 3 (3%) | 1 (2%) | 0.99 |  | 0 (0%) | 1 (5%) | - | |
| Coronary artery disease | 10 (10%) | 2 (4%) | 0.34 |  | 6 (15%) | 2 (10%) | 0.60 | |
| Myocardial infarction | 0 (0%) | 0 (0%) | - |  | 0 (0%) | 0 (0%) | - | |
| Valvular heart disease | 2 (2%) | 0 (0%) | 0.55 |  | 1 (2%) | 0 (0%) | - | |
| Cardiomyopathy | 1 (1%) | 0 (0%) | 0.99 |  | 0 (0%) | 0 (0%) | - | |
| Cardiac arrhythmia | 8 (8%) | 4 (8%) | 0.99 |  | 4 (10%) | 2 (10%) | 0.98 | |
| Congestive heart failure | 5 (5%) | 0 (0%) | 0.17 |  | 3 (7%) | 0 (0%) | - | |
| Arthritis | 28 (27%) | 10 (20%) | 0.43 |  | 11 (27%) | 6 (29%) | 0.89 | |
| Metabolic syndrome | 9 (9%) | 11 (22%) | **0.04** |  | 4 (10%) | 4 (19%) | 0.36 | |
| Smoker | 5 (5%) | 1 (2%) | 0.66 |  | 2 (5%) | 1 (5%) | 0.98 | |
| Chronic kidney disease | 3 (3%) | 1 (2%) | 0.66 |  | 2 (5%) | 1 (5%) | 0.67 | |
| Dialysis | 0 (0%) | 0 (0%) | - |  | 0 (0%) | 1 (1%) | - | |
| [Gastroesophageal reflux disease](http://en.wikipedia.org/wiki/Gastroesophageal_reflux_disease) | 36 (36%) | 9 (18%) | **0.04** |  | 11 (27%) | 4 (19%) | 0.48 | |
|  |  |  |  |  |  |  |  | |
| **Labs** |  |  |  |  |  |  |  | |
| Creatinine | 0.86 ± 0.28 | 0.84 ± 0.15 | 0.67 |  | 0.84 ± 0.25 | 0.85 ± 0.16 | 0.90 | |
| [Blood urea nitrogen, mmol/L](http://labtestsonline.org/understanding/analytes/bun/tab/test) | 15.5 ± 6.5 | 15.5 ± 5.6 | 0.99 |  | 15.4 ± 6.2 | 17.1 ± 7.0 | 0.34 | |
| Glomerular filtration rate | 58.1 ± 8.4 | 59.8 ± 0.9 | 0.23 |  | 59.1 ± 2.5 | 59.6 ± 1.3 | 0.50 | |
| Hemoglobin, gm/dL | 13.4 ± 1.4 | 13.6 ± 1.5 | 0.32 |  | 13.4 ±1.4 | 13.5 ± 1.7 | 0.80 | |
| Mean blood pressure, mmHg | 92.0 ± 14.1 | 89.9 ± 9.5 | 0.35 |  | 89.8 ±12.1 | 90.0 ± 10.7 | 0.96 | |
| Bilirubin, mg/dL | 0.45 ± 0.23 | 0.45 ± 0.19 | 0.99 |  | 0.44 ± 0.23 | 0.41 ±0.18 | 0.56 | |
| [Alanine aminotransferase](http://labtestsonline.org/understanding/analytes/alt) | 31.3 ± 34.5 | 37.3 ± 26.3 | 0.28 |  | 37.7 ± 50.5 | 28.6 ±17.7 | 0.46 | |
| Aspartate aminotransferase | 28.8 ± 29.6 | 31.4 ± 16.6 | 0.56 |  | 34.6 ± 44.1 | 27.0 ±13.1 | 0.91 | |
| Descriptive characteristics reported as mean and ± standard deviation or count (%)  ^a^ median (quartile 1 , quartile 3)  ^b^ p-values result from either one-way ANOVA or Fisher's exact test  ^c^ p-values result from linear mixed model or generalized estimating equation | | | | | | | |  |
